# Prévalence de l'utilisation des anti-inflammatoires non stéroïdiens chez les femmes enceintes à Lubumbashi (République Démocratique du Congo)

**DOI:** 10.11604/pamj.2014.18.132.4091

**Published:** 2014-06-11

**Authors:** Arsène Tshikongo Kabamba, Laurent Kwete Shamashanga, Jean-Jacques Mulubwa Mwaba, Christian Busambwa Kasongo, Albert Otshudi Longanga, Zet Kalala Lukumwena

**Affiliations:** 1 Faculté des Sciences Pharmaceutiques, Université de Lubumbashi, Lubumbashi, République Démocratique du Congo; 2 Université Libre de Bruxelles (ULB), Bruxelles, Belgique; 3 Faculté de Médecine Vétérinaire, Université de Lubumbashi(Unilu), Lubumbashi, République Démocratique du Congo

**Keywords:** AINS, femme enceinte, effets secondaires, automédication, NSAIDs, expectant mother, side effects, self-medication

## Abstract

Malgré l'importance de leurs effets secondaires sur la femme enceinte et le fœtus, les anti-inflammatoires non stéroïdiens (AINS) continuent à être largement utilisés par les femmes enceintes. Leur rapport bénéfice-risque n'est pas toujours bien évalué en pratique courante. L'objectif de ce travail est d’évaluer l'usage des AINS chez la femme enceinte, de discuter les risques potentiels encourus aussi bien par la mère que par le fœtus, et enfin d'en tirer des recommandations éventuelles à la femme enceinte. Du 22 août au 11 septembre 2012, une enquête a été menée auprès de 145 femmes enceintes suivies en consultation prénatale à l'Hôpital Sendwe. Un formulaire reprenant les informations sur les AINS consommés a été remis à chacune d'elle. Les résultats montrent que 75% des femmes interrogées reconnaissent avoir consommé des AINS surtout en automédication et principalement pendant les deux derniers trimestres de la grossesse pour soulager des douleurs d'origines diverses. Cette étude montre que des efforts restent encore à déployer à Lubumbashi afin de combattre l'utilisation des AINS surtout en automédication chez la femme enceinte.

## Introduction

Depuis le drame de la thalidomide en 1961, l'exposition d'une grossesse à des agents exogènes suscite de vives inquiétudes partagées par le grand public et le corps médical [1]. Or, au cours d'une grossesse connue, des thérapeutiques peuvent être nécessaires, voire indispensables au bon équilibre de la mère. La question du rapport bénéfice/risque pour la mère comme pour l'embryon ou le fœtus ne semble pas toujours aussi évidente à résoudre et les mises en garde souvent non spécifiques des notices des médicaments peuvent paraitre difficiles à interpréter [2–5]. Affirmer l'absolue innocuité d'un médicament est quasiment impossible. Par contre, il est souvent facile d'avoir une attitude prudente dans le choix du médicament pendant la grossesse [6]. Les AINS sont largement utilisés dans la population générale pour soulager les douleurs d’étiologies variées. Certaines précautions s'imposent quand à leur utilisation chez la femme enceinte, dépendant du stade de la grossesse, de l'indication thérapeutique et de la durée de traitement [7, 8]. Pour atteindre les objectifs de notre étude, nous avons réalisé différentes tâches notamment le recensement de différents AINS prescrits ou consommés en automédication pendant la grossesse et la maladie diagnostiquée à l'Hôpital Sendwe, la vérification du respect de leurs contre-indications, la diffusion de l'information sur les risques pour la mère et le fœtus liés à la prise des AINS par la femme enceinte.

## Méthodes

Notre étude est de type transversal. Elle vise à analyser l'utilisation des AINS chez les femmes enceintes à différentes périodes de la grossesse. Pour ce faire, un questionnaire a été rédigé et destiné aux femmes enceintes suivies en consultation prénatale à l'Hôpital JASON SENDWE. Celui-ci est un Hôpital de référence situé dans la ville de Lubumbashi. Il comprend en son sein un département de protection maternelle où les prescriptions médicales sont assurées par les infirmières et aussi par un médecin consultant. Cette étude a été réalisée du 22 août au 11 septembre 2012. Cent quarante-cinq femmes enceintes ont été invitées à répondre à un questionnaire reprenant les informations sur la femme enceinte, les AINS utilisés sur prescription ou en automédication. Parmi les informations concernant la femme enceinte, nous avons noté le nom et l'adresse de résidence, l’âge de la femme enceinte, l’âge de la grossesse, les AINS utilisés sur prescription ou en automédication ainsi que les différentes plaintes pouvant justifier leur utilisation.

## Résultats

Cent quarante-cinq femmes enceintes ont participé à notre enquête. Elle consistait à étudier l'utilisation des AINS pendant la grossesse par les femmes enceintes suivies en CPN à l'Hôpital Jason Sendwe de Lubumbashi. Une étude comparative de données sociodémographiques n'a pas été réalisée dans le cadre de cette enquête, le nombre effectif des femmes enceintes recensées étant très différent dans les différents groupes examinés (tranche d’âge, niveau d’étude, occupation professionnelle ..) (Tableau 1).

**Tableau 1:** Caractéristiques sociodémographiques des femmes enceintes interrogées à l'Hôpital SENDWE

Caractéristiques	Effectifs	Pourcentage
**Tranches d’âges**		
16-20	22	15,1
21-30	84	57,9
31-40	39	26,8
**Niveau d’études**		
Primaire	15	10,3
Secondaire	97	66,8
Supérieur	33	22,7
**Occupation professionnelle**		
Ménagères	17	11,7
Elèves	3	2
Etudiantes	5	3,4
Secteur informel	108	74,4
Salariées	12	8,2
**Age de la grossesse**		
Premier trimester		
16-20	9	6,2
21-30	19	13,1
31-40	1	0,7
**Deuxième trimester**		
16-20	13	9
21-30	32	22
31-40	4	2,8
**Troisième trimester**		
16-20	11	7,6
21-31	39	26,9
31-40	17	11,7

**Figure 1:**
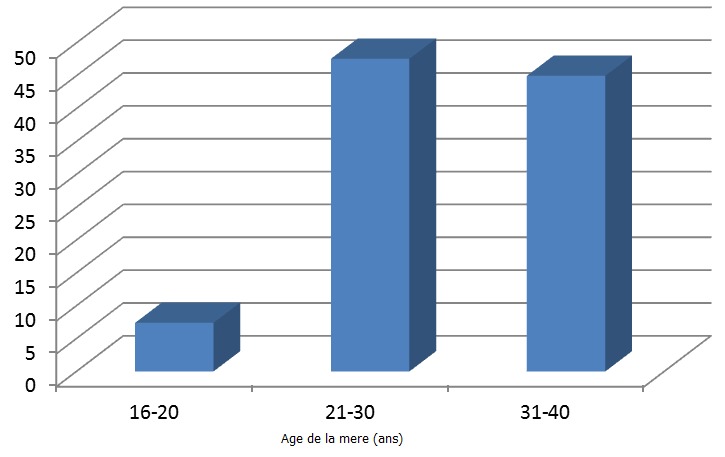
Taux de consommation des AINS par rapport à l’âge de la femme

**Figure 2:**
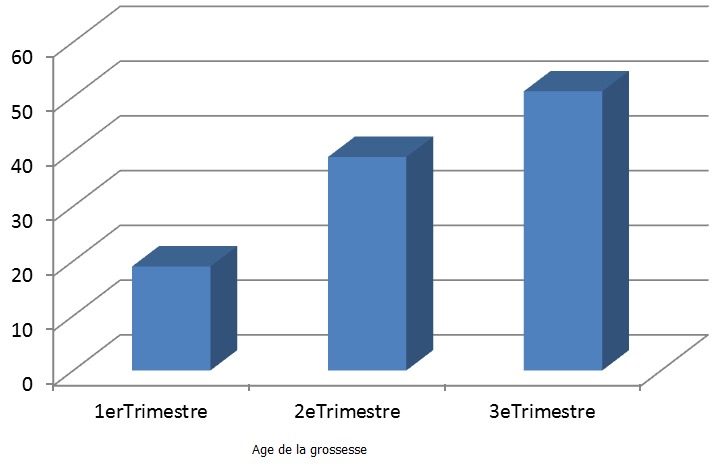
Taux de consommation des AINS par rapport à l’âge de la grossesse

**Figure 3:**
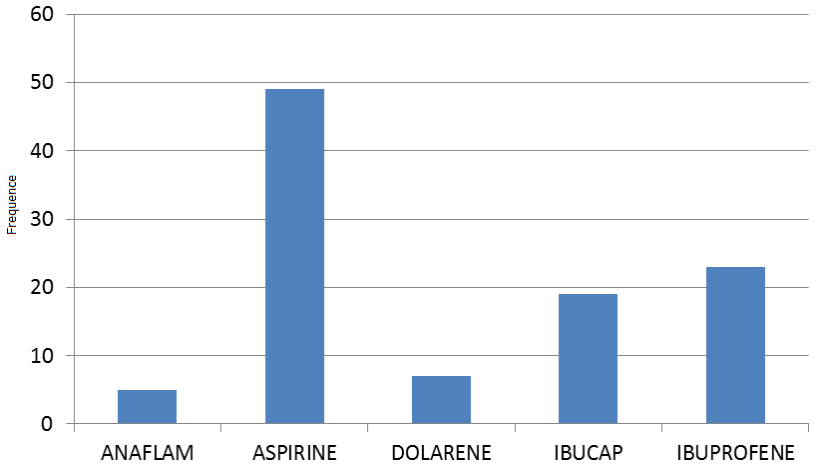
Fréquence de consommation des AINS

Sur les 145 femmes interrogées, 109 d'entre elles (75%) ont reconnu avoir consommé les AINS soit sur prescription médicale soit surtout en automédication. La plupart de prescripteurs concernés étaient des infirmiers. C'est dans la tranche d’âge de 21 à 30 ans (Figure 1) que nous retrouvons le taux de consommation d'AINS le plus élevé (47,6%). Au cours du troisième trimestre et dans une moindre mesure dans le courant du deuxième trimestre de la grossesse (Figure 2) nous avons enregistré les taux de consommation d'AINS les plus élevés. L'aspirine (acide acétylsalicylique, 49%) et l'ibuprofène (23%) sont les AINS les plus consommés par les femmes enceintes interrogées (Tableau 2, Figure 3).

**Tableau 2:** Les AINS consommés par les femmes enceintes interrogées à l'Hôpital SENDWE

AINS (Anti-Inflammatoire non Stéroïdien)	Fréquence de consommation
Anaflam^®^ (Ibuprofen + Paracétamol)	5%
Aspirine^®^ (Acide acétylsalicyque)	49%
Dolarene^®^ (Carisoprodol + Diclofenac)	7%
Ibucap^®^ (Ibuprofene +Paracétamol + Caféine)	19%
Ibuprofene	23%

## Discussion

Nos analyses ont révélés un taux de consommation d'AINS le plus élevé (47,6%) dans la tranche d’âge de 21 à 30 ans (Figure 1). Ceci est vraisemblablement dû au fait que la plupart de femmes enceintes dans cette tranche d’âge sont des femmes primigestes (Tableau 3), peu informées sur les effets secondaires d'AINS qu'elles consomment préférentiellement en automédication. Les raisons de cette automédication sont nombreuses, entre autres le coût élevé des consultations médicales particulièrement les consultations spécialisées, les refus des prescripteurs et l'utilisation courante d'AINS par les femmes en période non gravidique.

**Tableau 3:** Distribution des femmes primigestes selon les tranches d’âge

Tranches d’âge	Effectifs	Pourcentage
**Premier trimestre**		
16-20	9	6,2
21-30	16	11
31-40	0	0
**Deuxième trimestre**		
16-20	13	9
21-30	21	14,5
31-40	3	2
**Troisième trimestre**		
16-20	11	7,6
21-30	27	18,6
31-40	0	0

Le troisième trimestre et dans une moindre mesure dans le courant du deuxième trimestre de la grossesse (Figure 2), ont été marqués par les taux de consommation d'AINS les plus élevés. Pourtant, c'est pendant cette période que l'utilisation d'AINS est formellement contre-indiquée chez la femme enceinte. La principale raison de cette consommation est le soulagement de la douleur d'origine diverse: céphalées, douleurs dentaires ou post-traumatiques, douleurs abdominales, courbatures, symptômes d'arthrite etc.

L'aspirine (acide acétylsalicylique, 49%) et l'ibuprofène (23%) sont les AINS les plus consommés par les femmes enceintes interrogées (Tableau 2, Figure 3). L'importante consommation de l'aspirine peut s'expliquer par le fait qu’à la différence des autres AINS, l'aspirine est dotée des propriétés à la fois anti-inflammatoire, antalgique et antipyrétique. De plus, sa disponibilité et son prix de vente relativement bas la place parmi les AINS les plus facilement accessibles sur le marché pharmaceutique congolais.

Pourtant, l'aspirine à l'instar d'autres salicylés, traverse facilement le placenta [5, 6, 9]. Le métabolisme fœtal étant immature, il existe un risque de concentrations toxiques. Nous savons par ailleurs que la prise prolongée d'aspirine durant le dernier mois de grossesse entraîne une fermeture prématurée du canal artériel du fœtus, et chez la femme enceinte de manière particulière ont été décrits une augmentation d'accouchements prématurés, un allongement du temps de travail et une exacerbation des saignements au moment de la délivrance. Certaines études évoquent même la possibilité de risque accru d'avortements spontanés à la suite de la consommation d'AINS [6, 10].

Le risque hémorragique tant fœtal que maternel est le plus souvent attribué à l'effet anti-aggrégant plaquettaire des AINS. Les AINS sont des inhibiteurs des cyclo-oxygénases. Ils inhibent la synthèse des prostaglandines pro-inflammatoires et celles qui sont impliquées dans la contraction de l'utérus et l'ouverture du col utérin. Cette inhibition peut être responsable d'effets vasoconstricteurs sur certains territoires, particulièrement le rein et l'appareil cardio-pulmonaire [4–11].

Au niveau rénal, cette inhibition provoque une insuffisance rénale fœtale ou néonatale, transitoire ou définitive, pouvant entraîner la mort. Au niveau cardio-pulmonaire, la constriction in utero du canal artériel peut provoquer une mort fœtale in utero, une insuffisance cardiaque droite et une hypertension artérielle pulmonaire parfois mortelle chez le nouveau-né [8]. Pour ces différentes raisons, les AINS sont à éviter au cours des deux premiers trimestres de la grossesse et ils sont formellement contre-indiqués durant le troisième trimestre de celle-ci.

Toutefois, si un effet antalgique est recherché chez la femme enceinte, le paracétamol pourrait être proposé à la place d'AINS. En effet, à l'inverse des AINS, le paracétamol n'affecte pas la fonction plaquettaire fœtale et ne constitue pas de risque élevé d'hémorragies en péri-partum. Utilisé depuis de nombreuses années chez un grand nombre de femmes enceintes, aucun effet tératogène ni foetotoxique ne lui a été imputé [7]. Ce dernier, en cas de surdosage, peut quand même poser des sérieux problèmes hépatiques chez la mère qu'au fœtus au deuxième trimestre de grossesse [9].

## Conclusion

Les AINS sont très largement utilisés par les femmes enceintes à Lubumbashi. Les résultats de l'enquête que nous avons réalisée à l'Hôpital SENDWE corroborent cette affirmation. Or, l'utilisation de ces médicaments n'est pas sans danger aussi bien pour la femme enceinte que pour l'embryon ou le fœtus. C'est pourquoi, nous pensons que le corps médical devrait accorder une attention particulière à la prise en charge médicale de la femme enceinte. L'information sur une utilisation raisonnée d'AINS pendant la grossesse devrait être encouragée. L'idéal serait de déconseiller catégoriquement la prise d'AINS à la femme enceinte. Toutefois, dans les situations où une prescription deviendrait inévitable et lorsque l'effet antalgique est recherché chez la femme enceinte, le prescripteur portera son choix en priorité sur le paracétamol, le seul analgésique connu à ce jour comme étant dépourvu d'effets tératogène et foetotoxique. Les femmes enceintes devraient être suffisamment informées sur les risques encourus et le danger de l'automédication. Un effort particulier devrait être focalisé sur les femmes primigestes dans la tranche d’âge de 21 à 30 ans.

## Annexes

**Annexe 1**: Modèle du questionnaire utilisé lors de notre étude à l'Hôpital Jason Sendwe (PDF - 241 Kb)
